# Comparison of *Dracocephalum Moldavica* Essential Oil with Chlorhexidine on Cariogenic Bacteria

**DOI:** 10.30476/dentjods.2023.97602.2020

**Published:** 2024-09-01

**Authors:** Bahareh Nazemisalman, MScD; Seyede Solmaz Taheri, Fatemeh Heydari, Alireza Yazdinezhad, Fakhri Haghi, Mahsa Shabouei Jam, Samira Basir Shabestari

**Affiliations:** 1 Dept. of Pediatric Dentistry, Dental School, Zanjan University of Medical Sciences, Zanjan, Iran; 2 Student Research Committee, Dept. of Biostatistics, School of Allied Medical Sciences, Shahid Beheshti University of Medical Sciences, Tehran, Iran; 3 Dentist, Faculty of Dentistry, Zanjan University of Medical Sciences, Zanjan, Iran; 4 Dept. of Pharmacognosy, School of Pharmacy, Zanjan University of Medical Sciences, Zanjan, Iran; 5 Dept. of Bacteriology, Dept. of Microbiology and Virology, School of Medicine Zanjan University of Medical Sciences, Zanjan, Iran; 6 Dept. of ENT and Head and Neck, Firoozgar Hospital, School of Medicine, ENT and Head and Neck Research Center, Iran University of Medical Sciences, Tehran, Iran

**Keywords:** Chlorhexidine, *Dracocephalum moldavica*, Cariogenic Bacteria, *Lactobacillus acidophilus*

## Abstract

**Statement of the Problem:**

Dental caries and periodontal disease are the most common oral problems. Chemical antibacterial agents often have side effects; thus, researchers have long been in search for organic and herbal products to prevent dental caries and periodontal disease.

**Purpose:**

The present study has aimed to assess the effects of *Dracocephalum moldavica* essential oil on *Streptococcus mutans*, *Streptococcus salivarius*, *Streptococcus sobrinus*, and *Lactobacillus acidophilus* compared to Chlorhexidine (CHX).

**Materials and Method:**

In this *in vitro* study, the plants were collected from Zanjan Province, Iran. Analysis of the essential oil was carried out by gas chromatography/mass chromatography. Micro-broth dilution and disc diffusion methods were used for assessment of the antimicrobial activity, and minimum inhibitory concentration (MIC) and minimum bactericidal concentration (MBC) were evaluated.

**Results:**

The mean diameter of the growth inhibition zones in the well plate method for *Dracocephalum moldavica* showed that it had greater antimicrobial activity against *Lactobacillus acidophilus* than others (*p* Value= 0.01). Furthermore, *Dracocephalum moldavica* had higher antimicrobial activity than CHX. The results of MIC and MBC showed that *Dracocephalum moldavica* had lower antibacterial activity than CHX.

**Conclusion:**

*Dracocephalum moldavica* essential oil demonstrated antibacterial properties against cariogenic bacteria. Given that other favorable properties of these essential oils are confirmed, they may be suitable for use as antibacterial agents in the formulation of oral healthcare products.

## Introduction

Dental caries and periodontal disease are common oral conditions caused by the activity of bacteria in dental plaque [ 1 ]. The oral cavity is inhabited by a variety of microbial species, and oral microbial flora is highly complex since about 500 species are present in the oral cavity [ 2 ].

Several antibacterial agents such as fluoride compounds, phenolic compounds, and antibiotics have been extensively used for inhibition of bacterial growth and proliferation in the oral cavity; however, use of these synthetic compounds often disrupts the normal microbial flora of the mouth and intestines and causes numerous side effects [ 3 - 6 ]. 

Chlorhexidine (CHX) is the gold standard antibacterial mouthwash regarding its wide spectrum antibacterial properties in different concentrations. Its continuous use is not recommended due to the metallic taste as well as the resultant tooth discoloration [ 7 ]. At present, emergence of antibiotic-resistant bacteria species is a global dilemma. Thus, researchers have been in search of medicinal plants as alternatives to chemical agents [ 7 ]. At present, 25% of all drugs marketed in the United States have plant origin and can serve as suitable alternatives to chemical products in many conditions. Botanical resources are extensive and evaluation of different plant species in terms of their therapeutic efficacy and antimicrobial properties may lead to discovery of efficient herbs [ 8 ]. Researchers have been in search of effective medicinal plants against oral pathogens, which could be safe for use in the clinical settings [ 9 - 16 ]. 

The Labiatae family is famous for the essential oils found in many of its members, which have been utilized an antioxidants and antibacterial agents. Labiatae, with its 46 genera and 410 species and subspecies, exhibits significant diversity and widespread distribution in Iran. *Dracocephalum moldavica* L., which is locally known as Baderashbo in Iran is an annual, herbaceous, balm-scented plant belonging to the Lamiaceae family [ 17 - 18 ]. It is an edible medicinal plant. In traditional medicine, aerial parts of the plant have been used as infusions as sedative, hypotensive, antiemetic, and carminative agents. In Poland, *Dracocephalum moldavica* have been used as a cure for snake bites [ 19 - 20 ]. 

Considering the limited number of studies on the effects of herbal essential oils on oral and dental pathogens, and the need for herbal products with the same efficacy and fewer side effects compared to chemical agents, this study aimed to assess the effects of *Dracocephalum moldavica* essential oil on the most common cariogenic bacteria in comparison with CHX.

## Materials and Method

The University Ethics Committee approved this experimental study (IR.ZUMS.REC.1399.158). The *Dracocephalum moldavica* plants were collected from Zanjan when they were fully flowering. Plant samples were prepared and a voucher specimen was placed in the herbatium of the Department of Pharmacognosy, Faculty of Pharmacy, Zanjan University of Medical Sciences.

The oil from the dried and ground aerial parts of the plant was extracted using hydro distillation for three hours with a Clevenger type apparatus. The oil was then dried with anhydrous sodium sulfate and stored in the dark at +4°C under nitrogen until analysis.

The oil was analyzed using gas chromatography-mass spectrometry with an Agilent gas chromatograph and an Agilent HP-7890A (Wilmington, USA) mass selective detector in the electron impact mode. The analysis was conducted under the same oven temperature conditions as described above for the gas chromatography (GC), using a HP-5MS 5% phenylmethyl siloxane capillary column (30m×0.25mm, 0.25μm film thickness; Restek, Bellafonte, PA) and He as the carrier gas with a flow rate of 1 ml/min and samples (1.0μl) were injected with a split ratio of 1:10. The identification of essential oil components was based on comparing their retention indices to n-alkanes and using computer matching with the Wiley libraries. Additionally, the fragmentation pattern of the mass spectra was compared to data published in the literature [ 21 ].

For assessment of viability, *Streptococcus mutans*, *Streptococcus sobrinus*, *Streptococcus salivarius*, and *Lactobacillus acidophilus* were selected as the most common pathogens causing dental caries from Pasteur Institute of Iran. Microorganisms selected for testing were microbiologically identified and stored at -80°C in brain heart infusion broth including 15% (V/V) glycerol. Before testing, suspension was transferred to nutrient broth (NB) and cultured overnight at 37°C. Next, 0.5 McFarland standard concentration of microbial suspension containing 1.5×10^8^ colony forming units (CFUs)/ mL of bacteria was prepared. Two-fold serial dilution of suspension in 0.1 w/v% peptone in distilled water was inoculated onto nutrient agar (NA) for assessment of viability. 

The antimicrobial activity of essential oils was assessed using a disc diffusion test. Briefly, using a sterile cotton swab, microbial suspensions with the aforementioned concentrations were spread on Mueller Hinton agar. Sterile paper discs (6 mm diameter) were soaked in 10, 15 and 20µL of each essential oil and placed on inoculated plates. The plates were kept at 4°C for two h and incubated at 37°C for 24h. The diameter of the growth inhibition zones was measured in millimeters. All tests were repeated three times. 

The minimum inhibitory concentration (MIC) and minimum bacterial concentration (MBC) of essential oils were determined using a microplate Alamar blue assay. Two-fold serial dilutions of essential oils were prepared using a 96-well microplate. Dimethyl sulfoxide (160µL), 20µL of essential oil, and 140µL of Mueller Hinton broth were added to wells in row A. Wells in rows B to H received 80µL of Mueller Hinton broth. Two-fold serial dilution of each essential oil was prepared in the plate. Twenty µL of microbial inoculum containing 1.5×10^8^ CFUs/mL of each microorganism and 20 µL of Alamar blue was added to the wells to indicate growth and proliferation of bacteria. Overnight culture was prepared in NB such that the final concentration of each inoculum contained approximately 5×10^5^ CFUs/mL. The concentration of each inoculum was confirmed by counting the viable bacteria in Trypticase soy agar plate. A control microbial susceptibility test was carried out using gentamycin as a standard antibacterial agent and dimethyl sulfoxide 0.1% as the negative control. Plates were incubated at 37°C and tested at 24 and 48h. Color change from blue to red was considered as an indicator of bacterial growth. MIC was considered as the concentration of essential oil (µL/mL) in the first well, which remained blue. To determine MIC and obtain MBC, 10µL of broth from each well was removed and inoculated onto Trypticase soy agar (TSA) plate. After overnight aerobic incubation at 37°C, viable bacteria were counted. The MIC was considered as the minimum concentration causing a significant reduction in viability of bacteria (>90%) and MBC was considered as the concentration of essential oil killing more than 99.9% of primary bacterial count. Each test for each concentration of essential oils and for each microorganism was repeated three times.

## Results

The essential oil of *Dracocephalum moldavica* was obtained using hydro-distillation. The essential oil yield in aerial parts of *Dracocephalum moldavica* was 1.12% (v/w). The essential oil volatile compounds of *Dracocephalum moldavica* was; their retention indices (RI), retention time (RT) and percentages were presented in Table 1. The HP-5MS column was used to arrange all the constituents in order of their elution. A total of 11 components were identified in the essential oil from the aerial part, which accounted for 96.23 percent of the total essential oil. The antimicrobial effect observed may be attributed to the chemical composition of *Dracocephalum moldavica*, specifically gerany1 acetate (an ester derived from geraniol), geranial, and neral (collectively known as citral) which have strong antibacterial properties and thermal stability (Table 1).

**Table 1:** Chemical composition of the essential oil from *Dracocephalum moldavica*

Number	Compound	Retention Indexes	Retention Time (min)	Composition (%)
1	Sabinene	970	973	1.14
2	Linalool	1102	1093	2.21
3	Nerol	1229	1231	0.93
4	Neral	1236	1239	15.41
5	Geraniol	1253	1258	24.33
6	Geranial	1271	1277	8.21
7	Carvacrol	1302	1306	1.12
8	Neryl acetate	1361	1367	3.37
9	Geranyl acetate	1384	1388	37.66
10	Methyl eugenol	1412	1417	0.88
11	β-caryophyllene	1424	1429	0.97

### Results of disc diffusion test

The mean diameter of the growth inhibition zone of *Streptococcus mutans* in millimeter (based on the results of disc diffusion test) for *Dracocephalum moldavica*, CHX %0.02, and Gentamycin was found to be 16, 20 and 13 mm, respectively. The mean diameter of the growth inhibition zone of *Streptococcus sobrinus* for *Dracocephalum moldavica*, CHX %0.02, and Gentamycin was found to be 10, 22 and 8 mm, respectively. The mean diameter of the growth inhibition zone of *Streptococcus salivarius* for *Dracocephalum moldavica*, CHX %0.02, and Gentamycin was found to be 20, 30 and 14mm, respectively. The mean diameter of the growth inhibition zone of *Lactobacillus acidophilus* for *Dracocephalum moldavica*, CHX %0.02, and Gentamycin was found to be 36,26 and 26 mm, respectively. The well plate method revealed that *Dracocephalum moldavica* exhibited greater antimicrobial activity against *Lactobacillus acidophilus* compared to other substances.

Additionally, *Dracocephalum moldavica* displayed higher antimicrobial activity than CHX. 

Results of micro-broth dilution: The MIC of *Dracocephalum moldavica* and CHX for *Streptococcus mutans* were resistant 0,0.0031μg/mL, respectively. The MIC of *Dracocephalum moldavica* and CHX for *Streptococcus sobrinus* was 0.25312, 0.0003μg/mL, respectively. The MIC of *Dracocephalum moldavica* and CHX for *Streptococcus salivarius* was 4.05,0.0007μg/ mL, respectively. The MIC of *Dracocephalum moldavica* and CHX for *Lactobacillus acidophilus* was 2.025, 0.0031μg/mL, respectively. The MIC results indicated that *Dracocephalum moldavica* had less effective antibacterial activity compared to CHX. 

## Discussion

The antibacterial effects of its essential oil were evaluated using micro-broth dilution and disc diffusion methods. As shown in Tables 2 and 3, *Dracocephalum moldavica* essential oil demonstrated antibacterial activity against *Lacobacillus acidophilus* more effectively than other substances. Furthermore, *Dracocephalum moldavica* exhibited higher antimicrobial activity than CHX. 

**Table 2:** The diameter of growth inhibition zone in millimeters

Bacteria	Gentamycin	*Dracocephalum moldavica*	Chlorhexidine
*Streptococcus mutans*	13	16	20
*Streptococcus sobrinus*	8	10	22
*Streptococcus salivarius*	14	20	30
*Lactobacillus acidophilus*	26	36	26

**Table 3:** Minimum inhibitory concentration (MIC) and Minimum bacterial concentration (MBC) P. values

Bacteria	MIC(µg/mL)/ MBC(pg/mL)	*Dracocephalum moldavica*	Chlorhexidine
*Streptococcus mutans*	MIC	-	0.0031
	MBC	-	0.0031
*Streptococcus sobrinus*	MIC	0.25312	0.0003
	MBC	0.5062	0.0015
*Streptococcus salivarius*	MIC	4.05	0.0007
	MBC	4.05	0.0015
*Lactobacillus acidophilus*	MIC	2.025	0.0031
	MBC	2.025	0.0031

Antimicrobial activity of herbal essential oils depends on their chemical composition. Analysis of essential oils of different medicinal plants by chromatography has revealed their constituents to be monoterpenes, sesquiterpenes, and other oxygen-containing compounds such as alcohols, aldehydes, esters, ethers, ketones, and phenols [ 12 ]. Researchers have shown that among these compounds, carvones from the ketones, and thymol and carvacrol from the group of isomeric phenols have shown antimicrobial activity. Chemical composition of essential oils of plants is variable and one compound may be dominant in each essential oil [ 12 ].

Maham *et al*. [ 18 ] evaluated the constituents of *Dracocephalum moldavica* essential oil and reported that terpenoids were the main constituents with citral accounting for the major part (31.14%). Numerous studies have reported that Citral exhibits notable antimicrobial activity against both Gram-positive and Gram-negative bacteria [ 19 , 22 - 23 ]. 

El-Baky *et al*. [ 23 ] evaluated the antibacterial activity of *Dracocephalum moldavica* essential oil using bioautography and reported that among its constituents, geraniol, neral, geranyl acetate, geranial, nerol, neryl acetate and methyl nerolate possesseed antibacterial activity. They also showed that *Dracocephalum moldavica* essential oil had similar antibacterial activity (MIC=0.07 mg/mL) against *S. aureus* and *Micrococcus luteus* Gram-positive and *Serratia marcescens* Gram-negative bacteria, which was concentration-dependent [ 23 ]. They concluded that *Dracocephalum moldavica* essential oil had lower but significant antimicrobial activity compared to chloramphenicol (MIC=0.02mg/mL). In this study *Dracocephalum moldavica* showed the highest effect on *L. acidophilus* and created a larger growth inhibition zone compared to CHX. Lee *et al*. [ 24 ] examined the antimicrobial impacts of *Dracocephalum moldavica* and showed that it had antimicrobial effects against *Streptococcus mutans*, which was in contrast to our findings. This difference in the results of the two studies may be due to variability in *Dracocephalum moldavica* species or *Streptococcus mutans* strains evaluated. Another study demonstrated that different species of this plant had antimicrobial effects on different bacterial strains such as *Streptococcus mutans*, *Bacillus subtilis*, and fungi, which was in line with our results [ 20 ]. Similarity between the results of the two studies may be attributed to using the same plant species and bacterial strains [ 24 - 25 ].The *Dracocephalum moldavica* L. essential oil demonstrated better antioxidant and antimicrobial activities compared with the *Dracocephalum moldavica* hydrolate against *Staphylococcus aureus*, *Escherichia coli*, *Salmonella Typhimurium*, and *Listeria monocytogene* [ 26 ].

## Conclusion

*Dracocephalum moldavica* essential oil demonstrated antibacterial properties against cariogenic bacteria. If the confirmed positive qualities of these essential oils are taken into account, they could be used as antibacterial agents in oral healthcare products like mouthwashes, toothpaste, or dental floss to make use of their antimicrobial properties. The efficacy of these essential oils must be further evaluated in future *in vitro* studies with more precise techniques and clinical trials to validate the results of this study. 

## References

[1] Manji F, Dahlen G, Fejerskov O ( 2018). Caries and periodontitis: contesting the conventional Wisdom on their Aetiology. Caries Res.

[2] Struzycka I ( 2014). The oral microbiome in dental caries. Pol J Microbiol.

[3] Lemos JA, Palmer SR, Zeng L, Wen ZT, Kajfasz JK, Freires IA, et al ( 2019). The biology of Streptococcus mutans. Microbiol Spectr.

[4] Megalaa N, Thirumurugan K, Kayalvizhi G, Sajeev R, Kayalvizhi EB, Ramesh V, et al ( 2018). A comparative evaluation of the anticaries efficacy of herbal extracts (Tulsi and Black myrobalans) and sodium fluoride as mouthrinses in children: A randomized controlled trial. Indian J Dent Res.

[5] Naiktari RS, Gaonkar P, Gurav AN, Khiste SV ( 2014). A randomized clinical trial to evaluate and compare the efficacy of triphala mouthwash with 0.2% chlorhexidine in hospitalized patients with periodontal diseases. J Perio Implant Sci.

[6] Houshmand B, Mortazavi H, Alikhani Y, Abdolsamadi H, Ahmadi Motemayel F ( 2011). In vitro evaluation of antibacterial effect of myrtus extract with different concentrations on some oral bacteria. J Mashhad Dent Sch.

[7] Cieplik F, Jakubovics NS, Buchalla W, Maisch T, Hellwig E, Al-Ahmad A ( 2019). Resistance toward Chlorhexidine in Oral Bacteria- Is there cause for concern?. Front Microbiol.

[8] Hyldgaard M, Mygind T, Meyer RL ( 2012). Essential oils in food preservation: mode of action, synergies, and interactions with food matrix components. Front Microbial.

[9] Nazemi Salman B, Sallah S, Abdi F, Salahi S, Rostamizadeh K, Basir Shabestari S ( 2021). The Comparison of antimicrobial effect of Nigella sativa Nanoparticle and Chlorhexidine Emulsion on the most common dental Cariogenicic Bacteria. Med J Islam Repub Iran.

[10] Badalamenti N, Maresca V, Di Napoli M, Bruno M, Basile A, Zanfardino A, et al ( 2022). Chemical composition and biological activities of prangos ferulacea essential oils. Molecules.

[11] Salman B, Mohammadi M, Yazdinejad A, Zeighami H, Mohammadi A, Basir Shabestari S ( 2022). Antimicrobial activity of silver nanoparticles synthesized by the green method using Rhus coriaria L. Extract against oral pathogenic microorganisms. Med J Islam Repub Iran.

[12] Ionela DC, Ion IB ( 2007). Plant products as antimocrobial agents. License.

[13] Balekundri A, Mannur V ( 2020). Quality control of the traditional herbs and herbal products: a review. Futur J Pharm Sci.

[14] Al-Mariri A, Safi M ( 2013). The Antibacterial Activity of Selected Labiatae (Lamiaceae) Essential Oils against Brucella melitensis. Iran J Med Sci.

[15] Moumni S, Elaissi A, Trabelsi, A ( 2020). Correlation between chemical composition and antibacterial activity of some Lamiaceae species essential oils from Tunisia. BMC Complement Med Ther.

[16] Ramos da Silva LR, Ferreira OO, Cruz JN, de Jesus Pereira Franco C, Oliveira Dos Anjos T, Cascaes MM, et al ( 2021). Lamiaceae Essential Oils, Phytochemical Profile, Antioxidant and Biological Activities. Evid Based Complement Alternat Med.

[17] Shuge T, Xiaoying Z, Fan Z, Dongqing A, Tao Y ( 2009). Essential oil composition of the Dracocephalum moldavica L from Xinjiang in China. Pharmacogn Res.

[18] Maham M, Akbari H, Delazar A ( 2013). Chemical composition and antinociceptive effect of the essential oil of Dracocephalum moldavica L. Pharm Sci.

[19] Wójtowicz A, Oniszczuk A, Oniszczuk T, Kocira S, Wojtunik K, Mitrus M, et al ( 2017). Application of Moldavian dragonhead (Dracocephalum moldavica L.) leaves addition as a functional component of nutritionally valuable corn snacks. J Food Sci Technol.

[20] Khodaei M, Amanzadeh Y, Faramarzi M, Pirali Hamedani M ( 2018). Chemical analysis and anti-bacterial effect of essential oil from three different species of Dracocephalum in Iran. Am J Essential oil Nat Products.

[21] Nazemi Salman B, Basir Shabestari S, Shaboyi Jam M, Alizadeh Tari S, Shirinbak I ( 2020). Periodontal parameters and oral hygiene in diabetic and nondiabetic adolescents in Zanjan. Med J Islam Repub Iran.

[22] Onawunmi GO ( 2008). Evaluation of the antimicrobial activity of citral. Lett Appl Microbiol.

[23] El-Baky HH, El-Baroty G ( 2008). Chemical and biological evaluation of the essential oil of Egyptian Moldavian balm. Adv Food Sci.

[24] Lee SB, Cha KH, Kim SN, Altantsetseg S, Shatar S, Sarangerel O, et al ( 2007). The antimicrobial activity of essential oil from Dracocephalum foetidum against pathogenic microorganisms. J Microbiol.

[25] Sonboli A, Gholipour A, Yousefzadi M ( 2011). Antibacterial activity of the essential oil and main components of two Dracocephalum species from Iran. Nat Prod Res.

[26] A´cimovi´c M, Šovljanski O, Šeregelj V, Pezo L, Zheljazkov VD, Ljujić J, et al ( 2022). Chemical composition, antioxidant, and antimicrobial activity of Dracocephalum moldavica L. Essential Oil Hydrolate Plants.

